# A Study on Subjective Symptoms and Plantar Temperature Imbalance in Lumbar Spinal Stenosis: A Preliminary Study

**DOI:** 10.7759/cureus.79388

**Published:** 2025-02-20

**Authors:** Hiroto Chikubu, Kazuhide Inage, Sumihisa Orita, Yasuhiro Shiga, Masahiro Inoue, Kohei Okuyama, Soichiro Tokeshi, Keisuke Shimizu, Miyako Suzuki-Narita, Seiji Ohtori

**Affiliations:** 1 Orthopedic Surgery, Graduate School of Medicine, Chiba University, Chiba, JPN; 2 Orthopedic Surgery, Chiba University, Chiba, JPN; 3 Orthopedic Surgery, Eastern Chiba Medical Center, Togane, JPN; 4 Future Medicine Education and Research Organization, Chiba University, Chiba, JPN; 5 Bioenvironmental Medicine, Graduate School of Medicine, Chiba University, Chiba, JPN; 6 Orthopedics, Chiba University Hospital, Chiba, JPN

**Keywords:** laminectomy, lumbar spinal stenosis, plantar temperature, skin blood flow, thermography

## Abstract

Background

Symptoms of lumbar spinal stenosis (LSS) are associated with changes in blood flow to lower limbs. These changes in blood flow can be perceived as temperature variations, which can be visualized using thermography. We compared the relationship between the subjective symptoms of LSS and temperature variations before and after surgical intervention and investigated the associated patient factors.

Methods

Patients who underwent laminectomy for LSS were included. Plantar temperature was measured preoperatively and postoperatively using thermography. Subjective symptoms and patient backgrounds were assessed through interviews and medical records. Temperature differences were evaluated using the parameter ΔT, defined as the temperature of the limb with stronger symptoms minus the temperature of the limb with weaker symptoms. The analyses were performed based on these parameters.

Results

The symptoms improved following laminectomy, and ΔT showed a significant increase. Pain and ΔT were positively correlated preoperatively, whereas ΔT and numbness and neuropathic pain scores were negatively correlated postoperatively. Cauda equina symptoms were associated with a decrease in ΔT both preoperatively and postoperatively, whereas age and disease duration were associated with a postoperative decrease in ΔT.

Conclusion

The postoperative increase in ΔT, resulting from the alleviation of nerve compression, suggests a relationship between nerve dysfunction and decreased temperature. The preoperative correlation between pain and ΔT may be attributed to vasodilation mediated by calcitonin gene-related peptide. The postoperative correlation between ΔT and neurological symptoms may reflect an association between the extent of nerve damage and reduced blood flow. In particular, cauda equina symptoms may lead to decreased blood flow through dysfunction of the S region, causing a relatively sympathetic-dominant state due to impaired parasympathetic function. Our findings suggest the potential of using temperature measurements to visualize and objectively evaluate subjective symptoms.

## Introduction

The primary clinical symptoms of lumbar spinal stenosis (LSS) are lower limb pain and numbness [1]. These subjective symptoms are typically evaluated using tools such as the visual analogue scale (VAS) and numerical rating scale that assess the intensity of symptoms, such as pain and numbness, defining the minimum as no symptoms and the maximum as the worst symptoms imaginable. However, patients often report difficulty determining the severity of their maximum symptoms. Moreover, discrepancies between patient symptom severity and imaging findings are frequently observed during LSS. For instance, one study reported no significant correlation between the severity of spinal canal stenosis on MRI and the intensity of preoperative lower limb pain [2]. The complexity of symptom assessment is further compounded by reports suggesting that central sensitization may contribute to LSS-related pain [3]. 

The symptoms caused by LSS are not only related to mechanical compression of the spinal canal but also to blood flow disturbances [4,5]. The ankle-brachial pressure index (ABI) is commonly used to evaluate peripheral artery disease. Patients with symptomatic LSS had lower ABI values than patients with asymptomatic LSS, indicating reduced blood flow to the lower limbs [6]. Furthermore, the prevalence of diabetes mellitus, a condition that causes microvascular obstruction and various other pathological conditions, is high in patients with LSS [7-9]. Consequently, peripheral circulatory disorders may also be associated with symptoms of LSS.

One role of blood circulation is thermoregulation. Therefore, changes in blood flow can be visualized using thermography [10]. Thermography is a noncontact, noninvasive tool that objectively evaluates temperature and has been used to study the relationship between back pain and temperature changes [11]. Additionally, back pain severity and plantar region temperature are positively correlated [12], suggesting a relationship between peripheral circulation and back pain symptoms.

The aim of this study was to investigate the relationship between subjective symptoms of LSS and plantar temperature, with the goal of determining whether symptoms such as pain and numbness can be objectively assessed. By exploring the relationship between temperature and subjective symptoms, we aimed to consider the connection between blood flow and these symptoms, which may provide insights into the necessary therapeutic interventions.

## Materials and methods

Study participants

This study included 41 patients who underwent laminectomies at Chiba University Hospital between July 2022 and September 2024. Patients with obstructive arteriosclerosis and chronic renal failure requiring dialysis and those who underwent spinal fusion or emergency surgery were excluded before obtaining consent. The study was conducted on 41 patients who provided informed consent after receiving an explanation of the study on the day of hospital admission.

Ethical considerations

This study was conducted according to the ethical principles outlined in the Declaration of Helsinki and was approved by the ethical committee of my institution. Written informed consent was obtained from all patients.

Study overview

This was a prospective, observational study. Bilateral plantar temperatures were measured and interviews were conducted on the day before and on the sixth day after surgery. Patient background data were collected from the medical records.

Temperature Measurement Method

The plantar temperature was measured using a thermal imaging camera (FLIR One; TELEDYNE FLIR, California, USA). Measurements were conducted in the morning after breakfast and before rehabilitation sessions. The measurements were taken in patient rooms maintained at a constant temperature of 25°C throughout the year. The calibration process is performed automatically.

**Figure 1:**
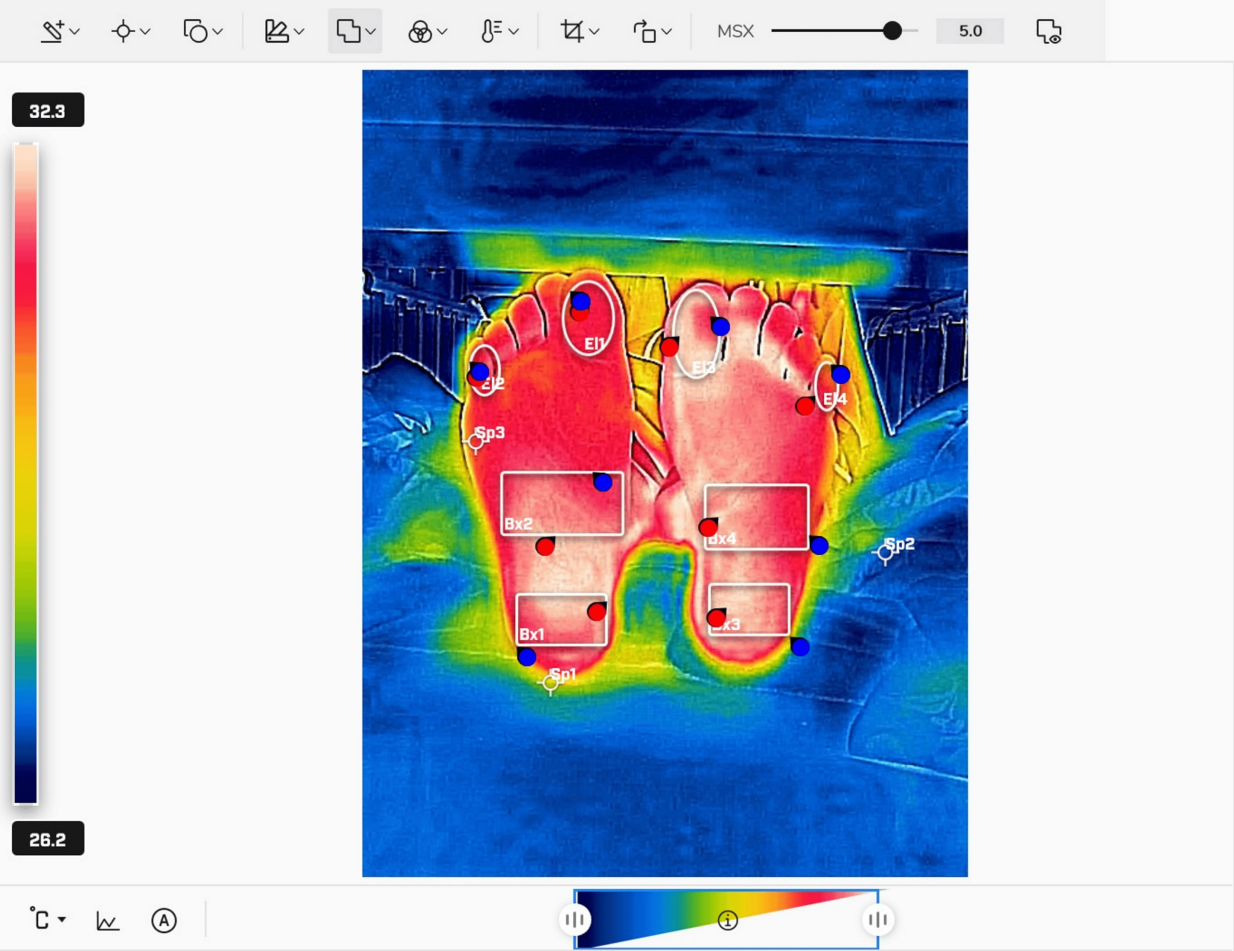
Thermographic image and temperature measurement method The average temperature of the regions enclosed by white circles and rectangles was calculated for each site, and the overall mean was defined as the plantar temperature Image taken by the authors

The patients exposed their feet to room temperature for 10 min before the measurements to standardize the conditions. The thermal imaging camera was positioned approximately 50 cm from the plantar surface, and temperatures were measured five times for both feet. The images were saved as still images for further analyses (Figure 1).

Temperature analysis was performed using the dedicated software, FLIR Thermal Studio software (Teledyne FLIR LLC, California, USA). The recorded data was transferred to a computer and analyzed using it. Temperatures were recorded at the big toe, small toe, heel, and plantar arches. The average of these values was defined as the plantar temperature. For each measurement set, the average temperature across the five readings was used for the analysis. Temperature differences between the two sides were expressed as ΔT, which was calculated as the following: ΔT = temperature of the side with stronger symptoms - temperature of the side with weaker symptoms.

The side with the strongest symptoms was determined based on patients' subjective reports. For patients who reported no lateral differences in symptoms, the side on which the symptoms first appeared was defined as the stronger side. In this study, none of the patients reported completely equal symptoms on either side.

Interview Content

The interview was conducted while the patient's feet were exposed to the atmosphere. Patients were interviewed regarding the duration of their condition, the side with the strongest symptoms, and the severity of their pain and numbness. After obtaining this information, the Douleur Neuropathique 4 (DN4) questionnaire was administered to assess neuropathic pain. Pain and numbness severity were assessed using the VAS. Data on the patient’s age, height, weight, and medical history were collected from interviews and medical records. The number of decompressed spinal segments was confirmed from the surgeon’s operative records, whereas information on medication use was obtained from the pharmacist’s records. In this study, cauda equina syndrome was defined as the presence of bladder and bowel dysfunction in addition to pain and numbness.

Analysis methods

Changes in ΔT before and after surgery were analyzed using the Wilcoxon signed-rank test. To analyze the relationship between ΔT and various factors pre- and postsurgery, the Mann-Whitney U test was used for binary explanatory variables, and the Spearman rank correlation coefficient was used for continuous variables. Statistical analyses were performed using EZR software, R Version 4.3.1 (R Foundation for Statistical Computing, Vienna, Austria). Statistical significance was set at p < 0.05.

## Results

Patient background

The patients’ backgrounds are summarized in Table 1, and their medication use is presented in Table 2. In the present study, the proportion of male patients was higher than that of female patients. Regarding medication use, 90% of the patients were taking medications, with mirogabalin being the most commonly used drug.

**Table 1:** Patient background BMI: body mass index; med: median; IQR: interquartile range; N: number

Factor		
Age (years, med (IQR))		73 (68,81)
Sex (male/female)		26 / 15
Height (cm, med (IQR))		162 (156, 170)
Weight (kg, med (IQR))		64.1 (57.8, 75)
BMI (kg/m^2^, med (IQR))		24.5 (22.5, 27)
Duration of the disease (months, med (IQR))		23 (11, 60)
Diabetes mellitus (N (rate))		11 (26.8%)
Smoking history (N (rate))		16 (39.0%)
Cauda equina symptom (N (rate))		20 (48.8%)
Number of decompression (N (rate))	1 vertebra	10 (24.4%)
	2 vertebrae	20 (48.8%)
	3 vertebrae	11 (26.8%)

**Table 2:** Medications used by patients NSAIDs: nonsteroidal anti-inflammatory drugs Dosage is shown as mean, minimum, and maximum

Factor	Number (rate)	Dose (mean (min, max))
Patients using drugs	37 (90.0%)	
Pregabalin	12 (29.3%)	164.6 (0, 300)
Mirogabalin	19 (46.3%)	12.9 (0, 30)
Tramadol	9 (22.0%)	95.8 (0, 150)
NSAIDs	12 (29.3%)	
Acetaminophen	10 (24.4%)	1087 (0,1500)
Neurotropin	3 (7.3%)	
Prostaglandin E1	13 (31.7%)	

Changes in ΔT before and after laminectomy

**Figure 2:**
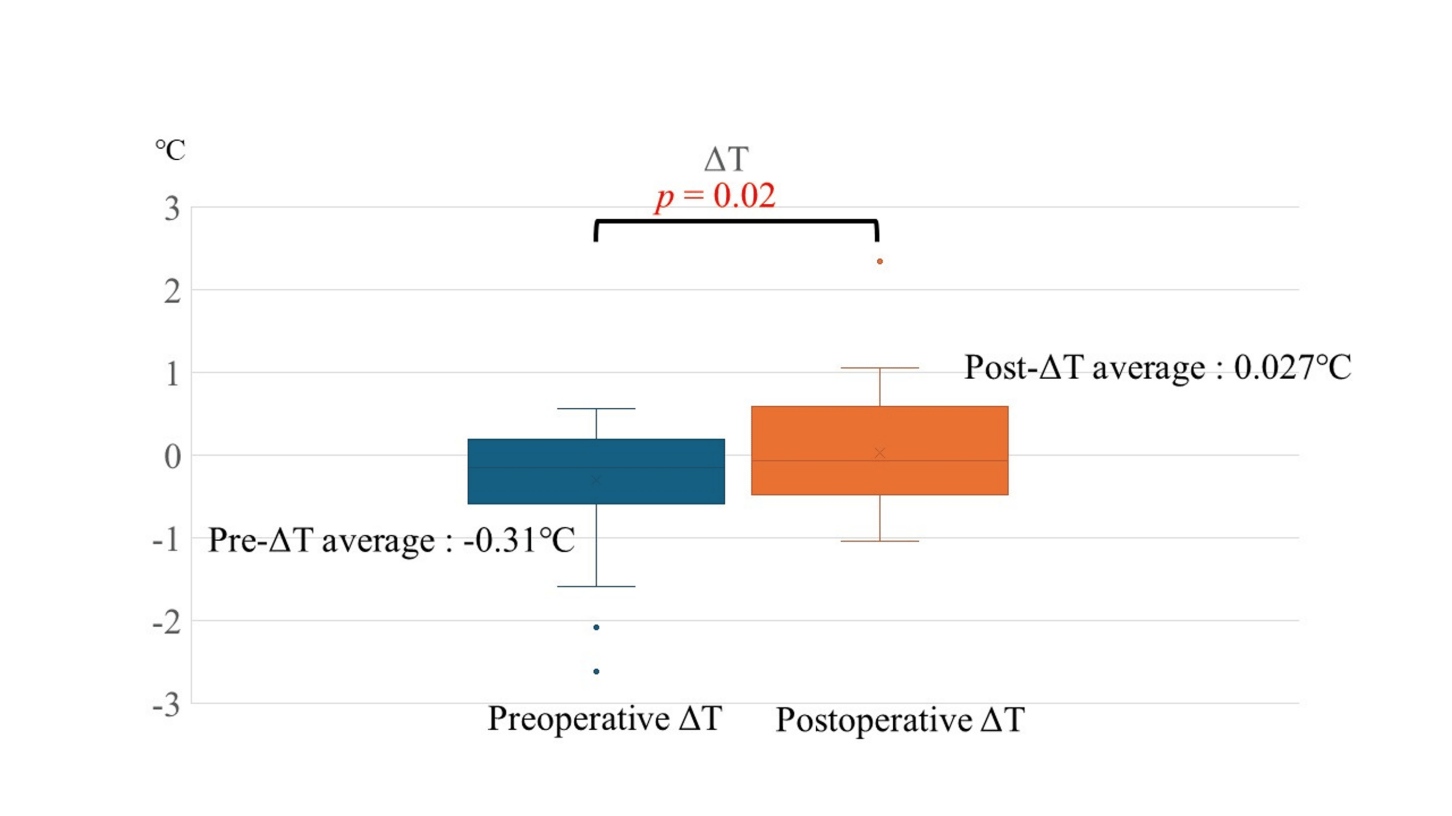
Comparison of preoperative and postoperative ΔT Statistical analysis: Wilcoxon signed-rank test. Statistically significant at p < 0.05 Image created by the authors

Changes in ΔT before and after laminectomy are shown in Figure 2. ΔT was significantly higher postoperatively compared to the preoperative values (p = 0.02, Figure 2). Additionally, the VAS scores for pain, numbness, and DN4 significantly reduced after surgery (p < 0.001; Table 3).

**Table 3:** Preoperative and postoperative subjective symptoms VAS: visual analogue scale; DN4: Douleur Neuropathique 4; IQR: interquartile range Statistical analysis: Wilcoxon signed-rank test. Statistically significant at p < 0.05

Factor	Preoperative (median,(IQR))	Postoperative (median, (IQR))	p-value
Pain VAS	8 (6,8)	3.5 (1,5)	<0.001
Numbness VAS	7 (5,8)	4.5 (0,6)	<0.001
DN4 questionnaire	4 (3,5)	3.5 (0,4)	<0.001

Factors associated with preoperative ΔT

**Figure 3:**
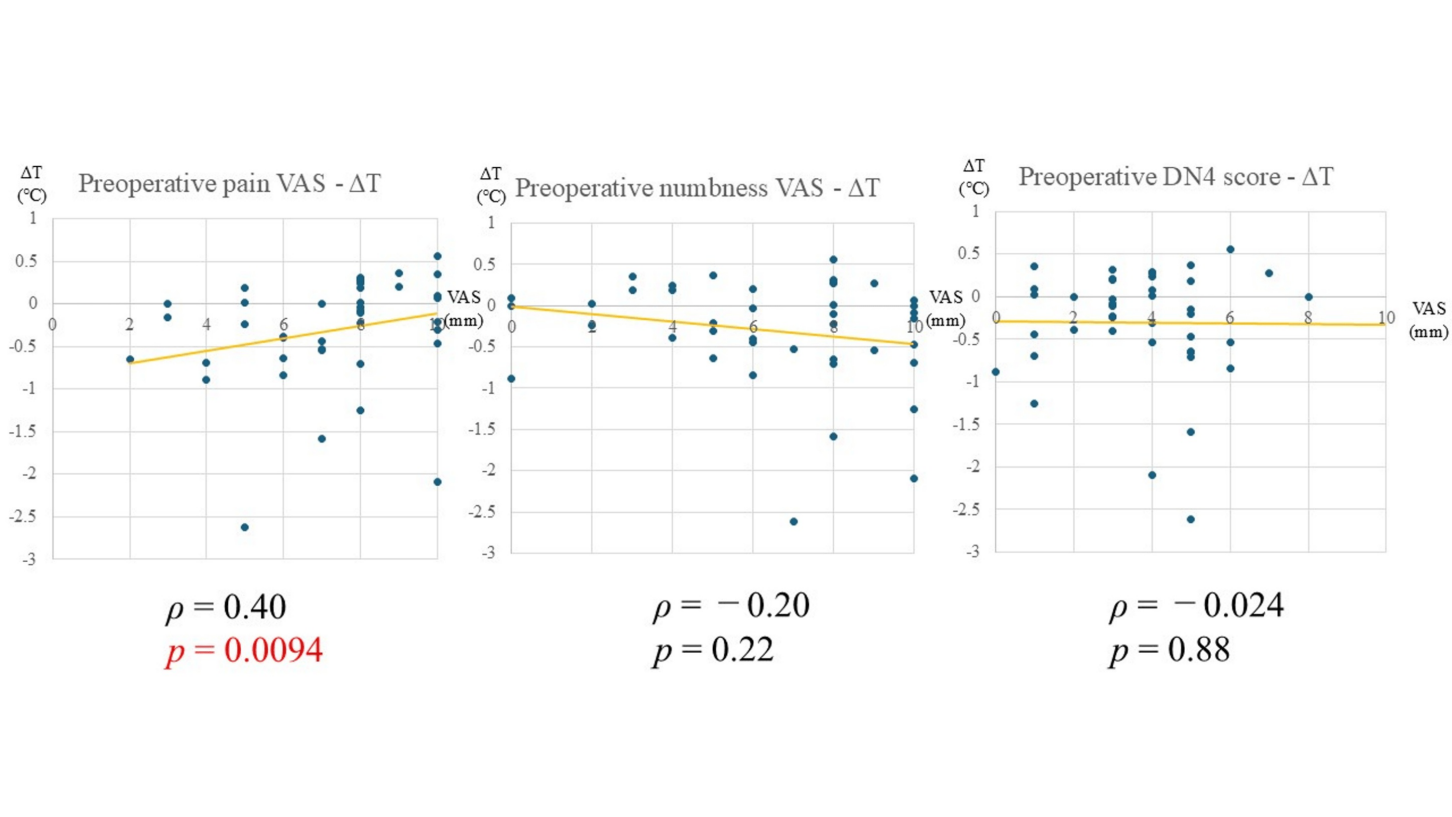
Relationship between preoperative subjective symptoms and ΔT VAS: visual analogue scale; DN4: Douleur Neuropathique 4 Statistical analysis: Spearman’s rank correlation coefficient. Statistically significant at p < 0.05 Image created by the authors

The analysis results of preoperative subjective symptoms and ΔT are shown in Figure 3. Preoperatively, pain VAS scores and ΔT (ρ = 0.40, p = 0.0094, Figure 3) were positively correlated. However, no significant correlation was found between ΔT and numbness or DN4 scores.

The analysis results of preoperative ΔT and patient factors are presented in Table 4. No factors were significantly associated with the temperature increase. However, patients with cauda equina symptoms showed significantly lower preoperative ΔT values (p = 0.03, Table 4).

**Table 4:** Relationship between preoperative ΔT and patient factors NSAIDs: nonsteroidal anti-inflammatory drugs; BMI: body mass index; PGE: prostaglandin E; DM: diabetes mellitus Statistical analysis: Spearman’s rank correlation coefficient for disease duration, age, and BMI. Other factors were analyzed using the Mann-Whitney U test. Statistically significant at p < 0.05

Factors	Analysis results		p-value
	ρ		
Duration of the disease	-0.16		0.32
Age	-0.23		0.14
BMI	0.11		0.48
	Yes or No	Median	
Cauda equina symptom	Yes	-0.42	0.030
	No	0.0015	
DM	Yes	-0.22	0.23
	No	-0.093	
Smoker	Yes	-0.24	0.70
	No	-0.095	
PGE	Yes	-0.31	0.062
	No	-0.041	
Mirogabalin	Yes	-0.080	0.65
	No	-0.22	
Pregabalin	Yes	-0.12	0.62
	No	-0.16	
Tramadol	Yes	0.020	0.20
	No	-0.23	
NSAIDs	Yes	0.052	0.12
	No	-0.23	
Acetaminophen	Yes	-0.07	0.85
	No	-0.21	

Factors associated with postoperative ΔT

**Figure 4:**
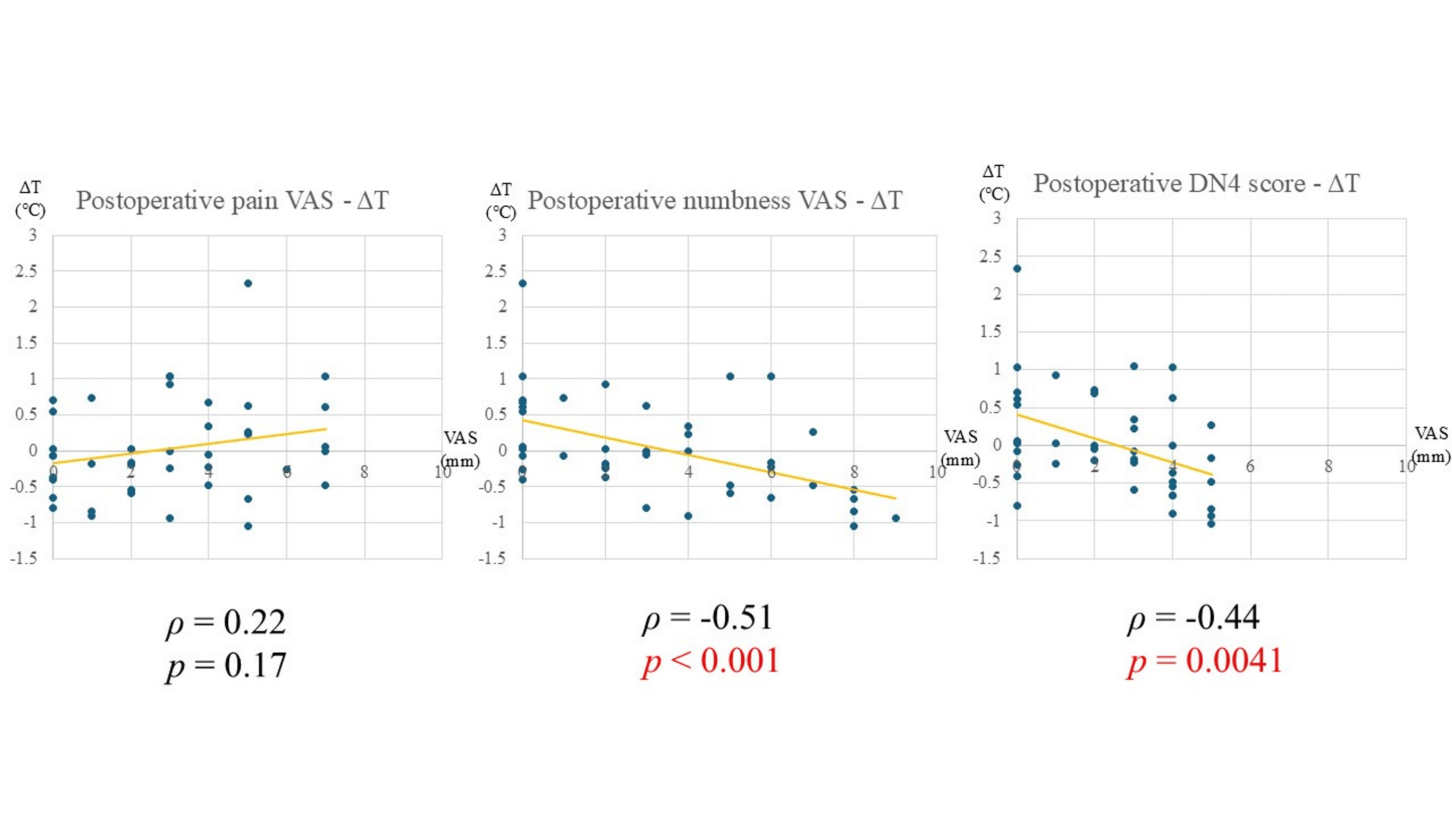
Relationship between postoperative subjective symptoms and ΔT VAS: visual analogue scale; DN4: Douleur Neuropathique 4 Statistical analysis: Spearman’s rank correlation coefficient. Statistically significant at p < 0.05

The analysis results of postoperative subjective symptoms and ΔT are shown in Figure 4. Postoperatively, ΔT showed a significant negative correlation with numbness VAS scores (ρ = -0.51, p < 0.001) and DN4 scores (ρ = -0.44, p = 0.0041, Table 5).

**Table 5:** Relationship between postoperative ΔT and patient factors NSAIDs: nonsteroidal anti-inflammatory drugs; BMI: body mass index; PGE: prostaglandin E; DM: diabetes mellitus Statistical analysis: Spearman’s rank correlation coefficient for disease duration, age, and BMI. Kruskal-Wallis test was used for the number of decompressed spinal segments. Other factors were analyzed using the Mann-Whitney U test. Statistically significant at p < 0.05

Factors	Analysis results		p-value
	ρ		
Duration of the disease	-0.31		0.045
Age	-0.36		0.022
BMI	-0.053		0.74
	Number of vertebrae	Median	
Number of decompression	1 vertebra	-0.005	0.59
	2 vertebrae	-0.18	
	3 vertebrae	-0.97	
	Yes or No	Median	
Cauda equina symptom	Yes	- 0.69	0.034
	No	-0.05	
DM	Yes	-0.070	0.44
	No	-0.058	
Smoker	Yes	-0.070	0.60
	No	0.043	
PGE	Yes	-0.19	0.09
	No	0.011	
Mirogabalin	Yes	-0.045	0.12
	No	-0.12	
Pregabalin	Yes	-0.015	0.74
	No	-0.070	
Tramadol	Yes	0.35	0.021
	No	-0.18	
NSAIDs	Yes	0.093	0.29
	No	-0.070	
Acetaminophen	Yes	-0.17	0.89
	No	-0.045	

The analysis results of postoperative ΔT and patient background factors are summarized in Table 5. Disease duration and age showed significant negative correlations with ΔT (disease duration: ρ = -0.31, p = 0.045; Age: ρ = -0.36, p = 0.022, Table 5). Additionally, patients with cauda equina symptoms exhibited significantly lower ΔT values postoperatively (p < 0.03, Table 5). Interestingly, ΔT was significantly higher in patients using tramadol (p = 0.021, Table 5).

## Discussion

Changes in ΔT following laminectomy

The significant postoperative increase in ΔT observed after laminectomy suggests that the temperature of the limb with stronger symptoms increased after decompression of the stenotic region. This increase in ΔT is likely due to improved blood flow resulting from the surgical intervention.

Pharmacological treatments, such as prostaglandin E1 derivatives, are commonly used for LSS. Yone et al. reported that patients who experienced vasodilation of the cauda equina vessels after the administration of prostaglandin E1 derivatives showed an improvement in subjective symptoms, whereas those without vascular changes did not experience symptom relief and subsequently underwent surgery [13]. These findings suggest that the symptomatic improvement achieved by laminectomy is not solely due to the mechanical decompression of stenosis but also involves enhanced blood flow. This dual mechanism of physical decompression and increased circulation highlights the critical role of blood flow in the management and symptomatology of LSS.

Factors associated with preoperative ΔT

A significant positive correlation was observed between pain VAS scores and ΔT preoperatively, consistent with previous reports [12]. In LSS, the mechanical compression of nerves results in nerve damage. This nerve damage increases the expression of calcitonin gene-related peptide (CGRP) [14]. CGRP, primarily expressed in C fibers and Aδ fibers, is often used as a marker for pain in basic research due to its involvement in pain signaling. However, its primary function is vasodilation [15]. Although various studies have examined the relationship between CGRP expression and pain intensity, it is generally considered that stronger pain leads to increased CGRP expression, which, in turn, promotes blood flow and results in elevated plantar temperatures.

No clear patient factors were associated with preoperative temperature elevation in this study. However, the presence of cauda equina symptoms was shown to significantly reduce ΔT. LSS with cauda equina symptoms involves multilevel dysfunction that particularly affects nerves in the S-region region. The S-region includes the parasympathetic nerves, and damage to these nerves can result in a relatively sympathetic-dominant state, leading to stronger-than-normal vasoconstriction. Consequently, cauda equina symptoms may contribute to reduced blood flow and a corresponding decrease in temperature.

Factors associated with postoperative ΔT

Postoperatively, ΔT showed no significant correlation with pain VAS scores but exhibited a significant negative correlation with numbness VAS and DN4 scores. This finding suggests that when temperature elevation in the symptomatic limb is not achieved, numbness and neuropathic pain tend to persist. The lack of temperature elevation indicates insufficient improvement in blood flow. After surgery, nerve decompression likely reduces the vasodilatory effects of CGRP, potentially revealing the underlying reduction in blood flow previously masked by CGRP-mediated vasodilation.

Peripheral circulatory and neurological conditions, such as diabetic neuropathy, are characterized by reduced blood flow and symptoms such as numbness and neuropathic pain. Diabetes causes microvascular damage by injuring endothelial cells, leading to peripheral nerve symptoms [16]. However, in the present study, diabetes and temperature reduction were not significantly associated. This suggests that microcirculatory impairment is linked to the severity of nerve damage, regardless of the presence of vascular endothelial dysfunction.

Postoperative ΔT was significantly associated with disease duration, age, cauda equina symptoms, and tramadol use. Longer disease duration likely results in accumulated nerve damage that cannot be fully reversed by decompression alone. Similarly, advanced age is associated with diminished nerve repair capabilities, which makes recovery unlikely. Prolonged disease duration was associated with residual postoperative numbness attributed to irreversible ischemic damage caused by chronic nerve compression [17]. Insufficient nerve repair owing to prolonged disease duration or advanced age may also impede microcirculatory recovery, leading to a lack of temperature elevation. Similar to preoperative temperature reduction, the presence of cauda equina symptoms may make it more challenging to resolve the relatively sympathetic-dominant state, resulting in persistent vasoconstriction and reduced blood flow.

Patients who preoperatively used tramadol exhibited significant postoperative temperature elevation. Although there are reports that excessive doses of tramadol suppress acetylcholine-induced vasodilation [18], no direct evidence suggests that tramadol itself induces vasodilation. Tramadol possesses serotonin-norepinephrine reuptake inhibition (SNRI) activity. Increased norepinephrine levels due to SNRI activity may act on α2-adrenergic receptors in the dorsal horn of the spinal cord, producing analgesic effects [19].

α2-adrenergic receptors, which are G protein-coupled receptors, inhibit cAMP production through Gi proteins, thereby suppressing pain transmission [20]. Additionally, clonidine, an α2-adrenergic receptor agonist, is effective for neuropathic pain, likely due to its peripheral vasodilatory effects via sympathetic inhibition [21,22]. Based on this, it is possible that norepinephrine, whose activity is improved by tramadol’s SNRI activity, acted on α2-adrenergic receptors to induce peripheral vasodilation, resulting in increased blood flow.

These findings highlight a potentially vicious cycle, in which nerve damage caused by reduced blood flow further exacerbates blood flow impairment, leading to postoperative residual neuropathy. The role of tramadol in improving microcirculation via its pharmacological effects may provide insights into therapeutic strategies to address such cases.

Discussion on the relationship between preoperative and postoperative ΔT and symptoms

In this study, pain exhibited a positive correlation with preoperative ΔT, whereas numbness and neuropathic pain demonstrated a negative correlation with postoperative ΔT. These findings suggest that the severity of pain is associated with vasodilation resulting from neurogenic inflammation caused by nerve impairment, while numbness and neuropathic pain may be influenced by microcirculatory disturbances affecting the nerves themselves, as well as imbalances in the autonomic nervous system.

Pain in LSS is not solely attributable to neuropathic pain but is also influenced by neurogenic inflammation induced by nerve compression. This inflammatory response is expected to subside following surgical decompression. In contrast, after surgery, as neurogenic inflammation diminishes, blood flow disturbances due to nerve damage become more apparent, potentially correlating with the severity of neuropathy.

Therefore, preoperatively, neurogenic inflammation leads to significant vasodilation, resulting in a positive correlation between pain and ΔT, while numbness and neuropathic pain, which are associated with blood flow disturbances, do not show a significant correlation. Postoperatively, the resolution of inflammation unmasks the underlying blood flow disturbances, leading to a negative correlation between ΔT and symptoms such as numbness and neuropathic pain, which reflect direct nerve damage.

These findings indicate that the influence of neurogenic inflammation may be a critical factor contributing to the discrepancy between subjective symptoms and ΔT observed preoperatively and postoperatively. Further studies incorporating larger cohorts and long-term follow-up are warranted to validate these observations and explore their clinical implications.

Limitations

This study had several limitations. The primary limitation is the limited number of cases. The sample size was insufficient to conduct a multivariate analysis of background factors; thus, only a univariate analysis was performed. Future studies should increase the number of cases to allow multivariate analysis and a more comprehensive evaluation of the relationship between temperature changes and background factors.

The observation period in this study was limited to the preoperative phase and up to six days postoperatively. Consequently, long-term symptom improvement and temperature changes could not be assessed. This also prevents the evaluation of interventions aimed at improving long-term blood flow. In particular, the effects of postoperative rehabilitation on lower-limb blood flow require further investigation. Long-term follow-up, including outpatient visits, is required to observe changes in temperature and symptoms over time. Temperature measurements were conducted at four points on the plantar surface, rather than covering the entire plantar area, owing to software limitations. Future studies should consider more detailed regions of interest settings to capture temperature changes across the entire plantar surface. However, the simplicity and feasibility of this method in the clinical setting must be considered. Developing an evaluation method that balances comprehensiveness and practicality remains a key goal. By addressing these limitations, future research could provide a more robust understanding of the relationship among temperature changes, symptoms, and interventions for LSS.

## Conclusions

This study demonstrated that higher preoperative temperatures were associated with increased pain, whereas lower postoperative temperatures were associated with a greater severity of numbness and neuropathic pain. These findings suggest the potential of using temperature measurements to visualize and objectively evaluate subjective symptoms.

Furthermore, this study highlighted the possible relationship between persistent postoperative numbness or neuropathic pain and peripheral blood flow, offering insights into the underlying mechanisms. Future research should investigate patient background factors associated with temperature reduction as well as the effects of medications and exercise on improving persistent symptoms and temperature changes after surgery. This can contribute to more effective management strategies for LSS.
